# Special Issue on Recent Advances in Sensors for Chemical Detection Applications

**DOI:** 10.3390/s25175422

**Published:** 2025-09-02

**Authors:** Michele Penza

**Affiliations:** Brindisi Research Center, Division of Technologies and Advanced Materials for Sustainable Manufacturing Industry, Department for Sustainability, ENEA—Italian National Agency for New Technologies, Energy and Sustainable Economic Development, I-72100 Brindisi, Italy; michele.penza@enea.it

**Keywords:** gas sensors, chemical sensors, sensor active materials, advanced functional nanomaterials, portable chemical sensor-systems, chemical sensor modeling, IoT devices, sensor engineering, chemical sensor applications, new concepts in chemical sensing

## Abstract

This Special Issue based on 15 articles/reviews focusses on low-cost sensor technology, gas sensors, chemical sensors, advanced active materials, sensing nanomaterials, sensor nodes, hardware innovation, data communication, system integration, sensor testing, functional characterization, sensor modeling, processing and correction algorithms, new sensing solutions, advanced proof of concepts, and chemical detection applications. Proper calibration techniques of chemical sensors have been explored, both in the laboratory and in field applications. Sensing solutions have been applied in the context of biochemical detection and gas monitoring.

## 1. Introduction

Chemical detection based on low-cost sensor technologies [1,2,3,4] has become increasingly popular for several emerging applications, such as industrial process control, chemical threat monitoring, green chemistry, environmental sustainability, smart cities, hydrogen economy, energy saving, wearable devices, IoT applications, public health protection, sustainable mobility, autonomous vehicles, and community sensing.

Functional materials [5,6,7,8,9] are cross-cutting technologies for chemical detection to provide advanced gas sensors at the laboratory level and real-world testing in many industrial applications. Low-power consumption, high-quality data, and optimal performance are some important parameters for a new generation of low-cost chemical sensors. Portable sensor systems and wireless sensor networks are typical approaches to monitoring chemical threats in long-term operation [10,11,12,13,14].

Current low-cost sensor technologies include numerous types of transducers, such as chemiresistors, electrochemical, transistor, optical, mass-sensitive, catalytic, and other hybrid configurations, evolving quickly with different open questions and considerable challenges, such as sensitivity, selectivity, stability, limit of detection, calibration, accuracy, and so on. Understanding the limitations and capabilities of current low-cost sensor technologies for chemical detection is a key issue for future applications.

This Special Issue of the *Sensors* (MDPI journal), titled “Recent Advances in Sensors for Chemical Detection Applications”, brings together fifteen articles presenting the recent developments in these areas. The publications range from advanced sensing materials, sensor devices, chemical detection, biosensing, calibration algorithms, new sensing solutions, proof of concepts, practical applications for environmental monitoring, and bio-sensing measurements. The featured research highlights significant advancements in biochemical sensing applied as smart technology for a sustainable future based on environmental, social, and governance methodology.

## 2. Overview of Published Papers

José Carlos Santos-Ceballos et al. (Contribution 1—Article) present a study devoted to the electrochemical modification of laser-induced graphene (LIG) with polyaniline (PANI), which led to the development of a chemo-resistive nanocomposite (PANI@LIG) for detecting ammonia levels at room temperature. The composite is characterized by Field Emission Scanning Electron Microscopy (FESEM), Fourier Transform InfraRed (FTIR), and Raman and X-ray Photoelectron Spectroscopy (XPS). Gas sensing mechanisms and functional tests have been discussed to fix sensing performance. The proposed sensor may offer higher response to ammonia, processing convenience, low-cost scalability, and a low limit of detection (LOD) of 2.38 ppb, as well as the sensor’s performance in real-world conditions, making this sensor a candidate for applications such as environmental monitoring and industrial safety. This work marks the first utilization of PANI@LIG for gas sensing and introduces a simple but effective approach for fabricating low-cost wearable gas sensors with high sensitivity and flexibility.

Rongqing Dong et al. (Contribution 2—Review) present a review devoted to conducting polymer-based gas sensors. Conducting Polymers (CPs) are promising materials for gas sensors due to their organic nature coupled with unique and versatile optical, electrical, chemical, and electrochemical properties. The fundamental gas sensing mechanisms in CPs-based sensors are elucidated, covering diverse transduction modes including electrochemical, chemo-resistive, optical, piezoelectric, and field-effect transistor-based sensing. Various types of conducting polymers employed in gas sensors, such as polypyrrole, polyaniline, polythiophene, and their composites, are introduced, with emphasis on their synthesis methods, structural characteristics, and gas-sensing response properties. Finally, the wide range of applications of these sensors is discussed, spanning industrial process control, environmental monitoring, food safety, biomedical diagnosis, and other fields, as well as existing issues such as long-term stability and humidity interference. The review has presented a comprehensive understanding of the CP-based sensors by examining their sensing mechanisms, sensitive materials, and device components. It also highlights future research directions, including device miniaturization, AI-assisted gas identification, multifunctional integrated sensing systems, and wearable and flexible sensor platforms.

Mahmoud Torkamani Cheriani et al. (Contribution 3—Review) present a review devoted to the plasma-treated nanostructured resistive gas sensors. Resistive gas sensors are among the most widely used sensors for the detection of various gases. In this type of gas sensor, the gas-sensing capability is linked to the surface properties of the sensing layer, and accordingly, modification of the sensing surface is of importance to improve the sensing output. Plasma treatment is a promising way to modify the surface properties of gas sensors, mainly by changing the amounts of oxygen ions, which have a central role in gas sensing reactions. After an introduction to air pollution, toxic gases, and resistive gas sensors, the main concepts regarding plasma are presented. Then, the impact of plasma treatment on the sensing characteristics of various sensing materials is also discussed. Generally, oxygen plasma causes the addition of surface oxygen functional groups on the sensor surface, and hence, the reactions between adsorbed gases with oxygen increase, leading to a higher sensing performance relative to pristine sensors. Also, exposure to other plasma atmospheres such as Ar or He causes the generation of oxygen defects, which act as favorable sites for oxygen adsorption and accordingly contribute to enhanced sensing performance. Different sensing materials such as metal oxides, TMDs, MXenes, CNTs, graphene, and CPs have been subjected to plasma treatment. In this regard, the combination of plasma exposure with other high irradiation techniques such as ion beams, electron beams, and gamma rays can lead to interesting sensing results.

Sanket Naresh Nagdeve et al. (Contribution 4—Review) propose a review devoted to the perspectives on the application of biosensors for the early detection of oral cancer. This study evaluates the significance of biomarkers and recent advancements in oral cancer detection, emphasizing cutting-edge electrochemical methods. The paper provides an epidemiological and etiological overview, outlining its clinical importance and reviewing the current state of the art in detection methods. Despite considerable progress, conventional methods exhibit limitations such as invasiveness, long wait times, and a lack of accuracy, creating a critical need for more robust technologies. This review emphasizes the significance of oral cancer biomarkers, which are considered promising cues for early detection, facilitating the development of innovative biosensing technologies. The review seeks to illuminate the recent advances in early detection and precision diagnostics, along with the usage of artificial intelligence strategies, ultimately contributing to significant progress in the battle against oral cancer. Integrating biomarkers and biofluids into the development and application of biosensors enhances the potential for accurate, reliable, and non-invasive oral cancer detection methods. Further research and development are essential to address challenges such as optimization for clinical settings, the validation of real-world applications, and integration into established diagnostic pathways. As the field of electrochemical biosensing continues to advance, the strategic integration of these biosensors into clinical practice holds promise to transform the landscape of oral cancer detection and management.

Chiheb Walleni et al. (Contribution 5—Article) propose a report on the synergistic effect of decorating nitrogen-doped reduced graphene oxide (N-rGO) with nickel oxide (NiO) nanoparticles for developing highly selective and sensitive chemi-resistive NO_2_ gas sensors. The N-rGO/NiO sensor was synthesized straightforwardly, ensuring uniform decoration of NiO nanoparticles on the N-rGO surface. Comprehensive characterization using SEM, TEM, XRD, and Raman spectroscopy confirmed the successful integration of NiO nanoparticles with N-rGO and revealed key structural and morphological features contributing to its enhanced sensing performance. As a result, the NiO/N-rGO nanohybrids demonstrate a significantly enhanced response five orders of magnitude higher than that of N-rGO toward low NO_2_ concentrations (<1 ppm) at 100 °C. In the first tests, the sensor showed a very high selectivity toward NO_2_ (the other gaseous species tested were CO_2_, ethanol, and NH_3_). Consequently, NiO NPs proved their potential for boosting the sensitivity of N-rGO toward NO_2_ gas, thanks to the p-p junctions created that facilitate carrier conduction, as explained by the underlying sensing mechanisms. Moreover, the present device has an outstanding performance, high sensitivity, and very low limit of detection (<1 ppb). The findings pave the way for integrating these sensors into advanced applications, including environmental monitoring and IoT-enabled air quality management systems. The nanomaterial presented robust performances, such as a high sensitivity and very low limit of detection, showing high prospects for being integrated in the next generation of advanced chemo-resistive sensors.

Bogdan-Catalin Serban et al. (Contribution 6—Article) propose a study on the ethanol vapor sensing performance of a resistive sensor that utilizes a quaternary nanohybrid sensing layer composed of holey carbon nanohorns (CNHox), graphene oxide (GO), SnO_2_, and polyvinylpyrrolidone (PVP) in an equal mass ratio of 1:1:1:1 (*w*/*w*/*w*/*w*). The sensing device includes a flexible polyimide substrate and interdigital transducer (IDT)-like electrodes. The sensing film is deposited by drop casting on the sensing structure. The morphology and composition of the sensitive film are analyzed using Scanning Electron Microscopy (SEM), Energy Dispersive X-ray (EDX) Spectroscopy, and Raman spectroscopy. The manufactured resistive device presents good sensitivity to concentrations of alcohol vapors varying in the range of 0.008–0.16 mg/cm^3^. The resistance of the proposed sensing structure increases over the entire range of measured ethanol concentration. Different types of sensing mechanisms are recognized. The decrease in the hole concentration in CNHox, GO, and CNHox due to interaction with ethanol vapors, which act as electron donors, and the swelling of the PVP are plausible and seem to be the prevalent sensing pathway. The hard-soft acid-base (HSAB) principle strengthens the proposed chemical analysis. Unlike conventional ethanol sensors, which primarily rely on metal oxides and rare elements, this innovative approach combines the synergistic properties of its components to enhance performance. CNHox provides high conductivity and porosity, improving electron transport and gas diffusion, while GO increases surface area and introduces functional groups that enhance ethanol interaction. SnO_2_ further strengthens ethanol adsorption and sensing response, and PVP ensures structural integrity and dispersion stability. Beyond performance, this sensor offers a cost-effective and environmentally friendly alternative to traditional designs.

Paniz Vafaei et al. (Contribution 7—Article) propose a study on low-power gas sensors that can be used in IoT (Internet of Things) systems, consumer devices, and point-of-care devices that will enable new applications in environmental monitoring and health protection. We fabricated a monolithic chemi-resistive gas sensor by integrating a micro lightplate with a 2D sensing material composed of single-layer graphene and monolayer-thick TiO_2_. Applying ultraviolet (380 nm) light with quantum energy above the TiO_2_ bandgap effectively enhanced the sensor responses. Low (<1 μW optical) power operation of the device was demonstrated by measuring NO_2_ gas at low concentrations, which is typical in air quality monitoring, with an estimated limit of detection less than 0.1 ppb. The gas response amplitudes remained nearly constant over the studied light intensity range (1–150 mW/cm^2^) owing to the balance between the photoinduced adsorption and desorption processes of the gas molecules. The rates of both processes followed an approximately square root dependence on light intensity, plausibly because the electron-hole recombination of photoinduced charge carriers is the primary rate-limiting factor. These results pave the way for integrating 2D materials with micro-LED arrays as a feasible path to advanced electronic noses. Finally, the authors developed a monolithic gas microsensor by integrating a UV microlight plate with a 2D sensing material made by CVD graphene and a less than a nanometer thick layer of TiO_2_ for advanced electronic noses with large sensor arrays.

Hsuan-Yu Chen et al. (Contribution 8—Article) propose a study on chemical analysis adopting a calibration curve to establish the relationship between the measuring technique’s response and the target analyte’s standard concentration. The calibration equation is established using regression analysis to verify the response of a chemical instrument to the known properties of materials that served as standard values. An adequate calibration equation ensures the performance of these instruments. There are two kinds of calibration equations: classical equations and inverse equations. For the classical equation, the standard values are independent, and the instrument’s response is dependent. The inverse equation is the opposite: the instrument’s response is the independent value. This study used measurement data sets from two kinds of humidity sensors and nine data sets from the literature to evaluate the predictive performance of two calibration equations. Four criteria were proposed to evaluate the predictive ability of two calibration equations. The study found that the inverse calibration equation could be an effective tool for complex calibration equations in chemical analysis. The precision of the instrument’s response is essential to ensure predictive performance. The inverse calibration equation could be embedded into the measurement device, and then intelligent instruments could be enhanced. The results of this study show that the inverse equation has excellent predictive performance for the calibration equation of the capacitive humidity sensor. The classical equation has better accuracy, and the inverse equation has better precision for the predictive performance of resistive humidity sensors. If the instrument response has good repeatability, the inverse equation performs excellently for the nine data sets collected in the literature. If the repeatability of the instrument response is poor, two calibration equations have similar predictive performance.

Trine Juul-Kristensen et al. (Contribution 9—Article) demonstrate a study devoted to the detection of the malaria-causing Plasmodium parasite in non-invasive saliva samples (N = 61) from infected individuals by combining a DNA-based Rolling-circle-Enhanced-Enzyme-Activity-Detection (REEAD) sensor system with a chemiluminescence readout that could be detected with an in-house-developed affordable and battery-powered portable reader. The authors’ team successfully transferred the technology to sub-Saharan Africa, where the malaria burden is high, and demonstrated a proof of concept in a small study (N = 40) showing significant differences (*p* < 0.00001) between malaria-positive individuals (N = 33) and presumed asymptomatic negative individuals (N = 7), all collected in Gabon. This is the first successful application of the REEAD sensor system for the detection of malaria in saliva in a high-epidemic area and holds promise for the potential future use of REEAD for malaria diagnosis or surveillance based on non-invasive specimens in sub-Saharan Africa. This study was conducted as a pilot investigation; more comprehensive field trials will be necessary to validate the diagnostic accuracy of the method, assessing its sensitivity and specificity.

Murugaiya Sridar Ilango et al. (Contribution 10—Article) present a study devoted to membrane permeability monitoring of the antipsychotic olanzapine using platinum black-modified electrodes. The blood–brain barrier (BBB) is key to the regular functioning of the central nervous system. The dysfunction of the BBB has been described in various neurological disorders, including schizophrenia. Schizophrenia (SCZ) is a chronic psychiatric disorder characterized by hallucinations, delusions, and negative symptoms. The Olanzapine (OLZ) drug is an electroactive species, and its levels can be monitored using electrochemical sensors. The detection of OLZ was demonstrated previously by using electrochemical sensors, and this technique can be used to monitor the levels of OLZ in real time. The challenge is to identify the permeability of OLZ through the BBB, so a replica model was designed with the BBB based on a Transwell membrane seeded with endothelial cells. A microfabricated electrode consisting of a 3 mm Au disk was modified with platinum black; this enables higher selectivity of electrochemical signals from OLZ. The dose–response of OLZ was characterized in a phosphate-buffered saline solution (10 mM, pH 7.4) by adding 20–200 nM (in steps of 20) of OLZ stock solution. The observed chronoamperometric electrochemical signals showed an increasing current at 0.45 V vs. Ag/AgCl with an increasing OLZ concentration. The controls for the experiments were performed in phosphate-buffered saline solution (10 mM, pH 7.4). The detection limit was calculated as 9.96 ± 7.35 × 10^−6^ nM from the calibration curve. The membrane permeability of the OLZ drug tested with five SCZ patients was monitored by studying the TEER measurements and permeability rate constant data. This study highlights the potential of electrochemical sensors for predicting human responsiveness to antipsychotic drugs. Platinum-black-modified electrodes were employed to detect the concentration of OLZ after their penetration through the BBB in various cell line models. The effective surface area of the platinum-black-modified electrodes is 5.23 × 10^−2^ ± 2.3 × 10^−3^ cm^2^, which is four times higher than the bare gold electrode, 3.81 × 10^−2^ ± 1.2 × 10^−3^ cm^2^. The dose–response of OLZ with platinum-black-modified electrodes was characterized using chronoamperometric electrochemical signals, which showed an increasing current at 0.45 V vs. Ag/AgCl with an increasing OLZ concentration.

Haixia Mei et al. (Contribution 11—Article) present a study devoted to research on the binary mixed VOCs gas identification method based on multi-task learning. Traditional volatile organic compound (VOC) detection models separate component identification and concentration prediction, leading to low feature utilization and limited learning in small-sample scenarios. Here, the authors realize a residual fusion network based on multi-task learning (MTL-RCANet) to implement component identification and concentration prediction of VOCs. The model integrates channel attention mechanisms and cross-fusion modules to enhance feature extraction capabilities and task synergy. To further balance the tasks, a dynamic weighted loss function is incorporated to adjust weights dynamically according to the training progress of each task, thereby enhancing the overall performance of the model. The proposed network achieves an accuracy of 94.86% and an R^2^ score of 0.95. Comparative experiments reveal that using only 35% of the total data length as input data yields excellent identification performance. Moreover, multi-task learning effectively integrates feature information across tasks, significantly improving model efficiency compared to single-task learning. In summary, the proposed method offers a new solution for gas detection tasks in fast detection and low-resource consumption scenarios, which shows great application potential. Future work can further optimize the network structure to enhance task collaboration, particularly in more complex gas mixtures or dynamic response scenarios.

Vadim Platonov et al. (Contribution 12—Article) present a report on the synthesis of perovskite-type Ba-doped LaFeO_3_ (La_1−x_Ba_x_FeO_3_, x = 0.00, 0.02, 0.04, and 0.06) nanofibers (NFs) using the electrospinning method. The synthesized La_1−x_Ba_x_FeO_3_ materials have a fibrous structure with an average fiber diameter of 250 nm. The fibers, in turn, consist of smaller crystalline particles of 20–50 nm in size. The sensor properties of La_1−x_Ba_x_FeO_3_ nanofibers were studied when detecting 20 ppm CO, CH_4_, methanol, and acetone in dry air in the temperature range of 50–350 °C. Doping with barium leads to a significant increase in sensor response and a decrease in operating temperature when detecting volatile organic compounds (VOCs). The process of acetone oxidation on the surface of the most sensitive La_0.98_Ba_0.02_FeO_3_ material was studied using in situ Diffuse Reflectance Infrared Fourier Transform Spectroscopy (DRIFTS) and Temperature-Programmed Desorption in combination with Mass Spectrometry (TPDMS). A mechanism for the formation of the sensor signal is proposed. The obtained materials were single phase, had an orthorhombic structure, and consisted of nanocrystallites with a size of about 14–16 nm. The introduction of barium led to the inhibition of crystallite growth during isothermal annealing and promoted an increase in the sensor response of the LaFeO_3_ nanofiber-based sensors toward VOCs. The La_0.98_Ba_0.02_FeO_3_ sample demonstrated the highest sensor response and a decrease in the operating temperature. The improvement in the gas-sensitive properties of the doped materials can be explained by the high catalytic activity of the surface of synthesized materials associated with the formation of oxygen vacancies, highly active iron cations (Fe^4+^), and coordinatively unsaturated cations (Fe^3+^). The mechanism of acetone oxidation on the sensor surface, studied using DRIFTS and TPD-MS methods, is assumed to have a multi-stage nature.

Alexey Vasiliev et al. (Contribution 13—Article) analyze the influence of micro hotplate size on the convective heat exchange of gas sensors. Usually, the role of convection in the heat exchange of gas sensors is not considered in thermal simulation models because of the complexity of the convection process. As a result, the contribution of this process to the overall heat loss of sensors remains without detailed analysis. The authors’ team analyzed convection issues in two groups of gas sensors: semiconductor and thermos-catalytic (calorimetric) sensors and, on the other hand, in the oxygen sensors of the thermomagnetic type. It is demonstrated that there is a critical size leading to the formation of convective heat exchange flow. Below this critical value, only thermal conductivity of ambient air, IR (infrared) radiation from the heated micro hotplate surface, and thermal conductivity of the micro hotplate supporting elements should be considered as channels for heat dissipation by the micro hotplate, and the contribution of free convection can be neglected. Similar results were obtained in the analysis of the behavior of thermal magnetic sensors of oxygen, which use paramagnetic properties of molecular oxygen for the determination of O_2_ concentration. In this case, the critical size of the sensor is also of significance; if the size of the magnetic sensor is much below this value, the oxygen concentration value measured with such a device is independent of the orientation of the sensor element. The results of the simulation were compared with the measurement of heat loss in micromachined gas sensors. The optimal dimensions of the sensor micro hotplate are given as a result of these simulations and measurements. The authors’ team investigated the influence of convection on the heat exchange processes of micro hotplates used in the fabrication of semiconductor and thermos-catalytic (calorimetric) gas sensors, as well as the heat exchange of thermal magnetic sensors of oxygen. The analysis was based on the consideration of the competition of convection flow and back diffusion. It was shown that there is a certain critical size of the micro hotplate. If the size of the micro hotplate d << d_cr_, the influence of convection heat exchange can be neglected, and only the thermal conductivity of air and of the elements of the sensor, together with the IR (infrared) radiation (if the sensor is heated up to a very high temperature), should be taken into account as channels of heat losses.

O. L. Gribkova et al. (Contribution 14—Article) report on optical ammonia sensors based on spray-coated polyaniline complexes with polysulfonic acids. The optical ammonia-sensing properties of water-dispersible polyaniline (PANI) complexes chemically synthesized in the presence of polysulfonic acids of different structures and chain flexibility were compared for the first time. Flexible-chain poly(styrene-4-sulfonic acid) and poly-(2-acrylamido-2-methyl-1-propanesulfonic acid), as well as semi-rigid-chain poly-4,4′-(2,2′-disulfonic acid)diphenylene-iso-phthalamide and rigid-chain poly-4,4′-(2,2′-disulfonic acid) diphenylene-tere-phthalamide (t-PASA) were used. The sensor films were prepared by a convenient and scalable method: spray coating of aqueous solutions on glass substrates. The optical response time and amplitude of the sensor films in the range of ammonia concentrations from 5 to 200 ppm were investigated. To overcome the influence of humidity and the presence of over-stoichiometric protons of the polyacid on the accuracy of ammonia determination, treatments of the films in aqueous solutions of NaCl, CaCl_2_, and BaCl_2_ were tested. The treatment in 1 M CaCl_2_ solution for all the PANI complexes results in a significant improvement in the response time, amplitude, and reproducibility. The films of PANI complexes with the flexible-chain polyacids have the highest response amplitude in the range of ammonia concentrations 5–25 ppm. PANI-t-PASA film demonstrated the best sensory properties at ammonia concentrations more than 50 ppm. FTIR spectroscopy showed that CaCl_2_ treatment results in cross-linking of sulfoacid groups from adjacent polyacid chains by Ca^2+^ ions. Thus, such a treatment results both in the neutralization of excessive protons and a significant reduction in the films’ swelling at high humidity. Among the films of the PANI complexes treated with CaCl_2_, the best sensory properties were demonstrated by the PANI-t-PASA film at ammonia concentrations more than 50 ppm. The films of PANI complexes give reproducible results at 2–4 reuses, the reversibility decreased at high ammonia concentration. Therefore, at this stage of the investigations, these films are preferably to be used as alarm detectors.

Anas Mohd Noor et al. (Contribution 15—Article) report on a wearable device for continuous and real-time monitoring of human sweat sodium. Wearable sweat-sensing devices hold significant potential for non-invasive, continuous health monitoring. However, challenges such as ensuring data accuracy, sensor reliability, and measurement stability persist. This study presents the development of a wearable system for the real-time monitoring of human sweat sodium levels, addressing these challenges through the integration of a novel microfluidic chip and a compact potentiostat. The microfluidic chip, fabricated using hydrophilic materials and designed with vertical channels, optimizes sweat flow, prevents backflow, and minimizes sample contamination. The developed wearable potentiostat, as a measurement device, precisely measures electrical currents across a wide dynamic range, from nanoamperes to milliamperes. Validation results demonstrated accurate sodium concentration measurements ranging from 10 mM to 200 mM, with a coefficient of variation below 4% and excellent agreement with laboratory instruments (intraclass correlation = 0.998). During physical exercise, the device measured a decrease in sweat sodium levels, from 101 mM to 67 mM over 30 min, reflecting typical physiological responses to sweating. These findings confirm the system’s reliability in providing continuous, real-time sweat sodium monitoring. This work advances wearable health-monitoring technologies and lays the groundwork for applications in fitness optimization and personalized hydration strategies. Future work will explore multi-biomarker integration and broader clinical trials to further validate the system’s potential. The microfluidic chip, fabricated using a water-washable resin and 3D printing technology, offers high resolution, rapid fabrication, and excellent hydrophilicity. Additionally, investigating the performance of the proposed device in larger-scale clinical trials could further validate its potential for real-world applications.

Finally, the research presented is pivotal in shaping the future of chemical sensing by means of advanced sensor systems and devices integrating innovative functional materials for advancements in biochemical applications.

## 3. Statistics and Trend Analysis

Trend analysis is reported in Table 1. The Special Issue offers an outstanding overview of recent advancements in biochemical sensors for detection applications. The statistics consisted of 15 published papers, including 3 reviews and 12 articles co-authored by 89 international scientists from 15 countries located in Europe, Africa, Asia, and North America. The total number of the rejected manuscripts is two.

The challenges of the Special Issue deal with advanced sensing materials (graphene-based materials, carbon nanomaterials and composites, metal oxide nanoparticles, heterostructures, perovskites, hybrid functional materials, and polymeric complexes) and related material processing (laser and plasma) for optimal biochemical sensing properties. Furthermore, the findings push the knowledge in the advanced transduction of biochemical sensing (electrochemical, chemo-resistive, optical, analytical, spectroscopic, microfluidic, micro-hotplate, thermal, biomarker, and test kit). The calibration of sensors, predictive performance, multi-task learning, and feature fusion have been explored as well.

Challenging applications have been addressed, such as sub-ppb detection of toxic gases and volatile organic compounds for environmental monitoring, malaria detection by fast test kits for in-field biosensing, schizophrenia by the biomarker of olanzapine using platinum electrodes, and sweat sodium measurement by microfluidic chip for wearable applications.

## 4. Summary and Conclusions

The work presented in this Special Issue reflects the drive to increase the knowledge in chemical sensors by applied research in advanced materials, sensing devices, new transducers, and practical applications. In this Special Issue, front-line scientists have been kindly invited to submit original research and review articles on exploring recent advances in sensors for chemical detection applications.

Potential topics included, but were not limited to, gas sensors, chemical detection, advanced materials for chemical sensing, novel gas sensor materials, sensor calibration, sensor systems, machine learning algorithms, wireless sensor networks, chemical threat monitoring, environmental measurements, sensors for smart city applications, sensors for environmental sustainability, sensors for energy applications, sensors for IoT applications, sensors for industrial applications, sensors for sustainable mobility, case studies of chemical detection campaigns, and new concepts and trends in chemical sensing.

The impactful achievements presented in this Special Issue are a valuable contribution to the theory and practice of chemical sensing. Additional development of these trends can be expected in the future to consolidate the development of a new generation of chemical sensors applied for practical applications.

## Figures and Tables

**Table 1:** Main features of the papers published in the Special Issue.

Paper	Type	Nr. of Authors	Corresponding Author Country	Other Authors’ Countries	Keywords
Contribution 1	Article	6	Spain	Spain	laser-induced graphene; polyaniline; gas sensing; RT ammonia sensing
Contribution 2	Review	7	China	China	conducting polymer; gas sensor; sensing mechanisms; environmental monitoring
Contribution 3	Review	2	Iran	Iran	plasma treatment; toxic gas; gas sensor; sensing mechanisms
Contribution 4	Review	3	USA	USA	biomarkers; biofluids; electrochemical sensors; molecular analytical techniques; diagnostic tools; commercial test kits
Contribution 5	Article	4	Spain	Spain; Tunisia	N-doped reduced graphene oxide; nickel oxide nanoparticles; sub-ppb NO_2_ sensing; gas sensing
Contribution 6	Article	9	Romania	Romania	ethanol sensor; holey carbon nanohorns; graphene oxide; swelling; HSAB (hard soft acid base) principle
Contribution 7	Article	7	Estonia	Estonia; Spain; Germany	gas sensor; NO_2_ gas sensing; micro-lightplate; graphene/TiO_2_ heterostructure
Contribution 8	Article	2	Taiwan	Taiwan	calibration; classical equation; inverse equation; predictive performance
Contribution 9	Article	13	Denmark	Denmark; Gabon; Germany	malaria; diagnosis; rolling circle amplification; saliva; topoisomerase 1
Contribution 10	Article	6	Israel	Israel	olanzapine; platinum black; blood–brain barrier; electrochemical sensors; schizophrenia
Contribution 11	Article	6	China	China	gas sensor; multi-task learning; mixed gases; feature fusion
Contribution 12	Article	5	Russia	Russia	Ba-doped LaFeO_3_; perovskites; semiconductor gas sensor; VOCs; DRIFTS (diffuse reflectance infrared Fourier transform spectroscopy); TPD-MS (temperature-programmed desorption in combination with mass spectrometry)
Contribution 13	Article	4	Russia	Russia	microheater; convective heat losses; thermal conductivity; Grashof number
Contribution 14	Article	6	Russia	Russia	polyaniline complexes; polyacid; spray coating; ammonia sensors; optical gas sensors
Contribution 15	Article	9	Malaysia	Malaysia; Japan	microfluidic chip; sweat sodium measurement; wearable device
Total Authors involved		89			
Total Author Countries		15			
Total Rejected Papers		2			
Total Published Papers		15			
